# Oil in Storms at Sea

**Published:** 1885-07

**Authors:** 


### Oil in Storms at Sea.

The Hydrographic Office of the Navy Department has for several months been engaged in collecting data to determine under what circumstances the use of oil is most efficacious in diminishing the danger of breaking seas during gales of wind. When sufficient data have been collected, it is proposed to issue a pamphlet giving such directions in regard to the use of oil as common experience of seamen may determine to be best.

The following are among the most striking of the accounts recently received:

In November, 1881, the steamship Venice, from Savannah to Europe with cotton, while running before a heavy northwest gale was boarded by a tremendous sea. The captain determined to heave to, and men were stationed to pour oil down the closet chutes forward and to throw waste, soaked in oil, to windward. The vessel came round without shipping any water. As she kept falling off, it was concluded to put her again before the sea, which was done without trouble, and it was found that she kept perfectly dry as long as the oil was used. Again, in January, 1884, while crossing the Atlantic to New York, after running before a northwest gale for some time, she was laid to without difficulty or danger by using oil in the manner stated.

Captain Ritchie, of the English steamer Fern Holme, while on his last voyage from Baltimore to Shields used oil bags while running before a west-southwest gale. He hung one over each side, just forward of the bridge, and they prevented the ship from taking water on deck.

First Officer W. Maltjen, of the German steamer Colon, in December, 1884, used oil bags with remarkable effect. Two bags filled with boiled oil were hung over the bow. The oil spreading over the surface prevented the waves from breaking, and the ship rode quite easily during the continuance of the gale.

Captain Jones, of the British steamer Chicago, while rescuing the crew of the brig Fedore, used oil with best results. It was blowing a heavy gale, with very high seas. The Chicago ran to windward of the Fedore, and during a lull, oil having been poured on the water, the port lifeboat was successfully launched and started. A can of oil was taken in the boat, and by using this the seas were kept down in the immediate vicinity, though they broke in masses of foam a short distance away. As the boat approached the Fedore, the crew of that vessel poured oil on the water, which so calmed the sea that the boat alongside and rescued the shipwrecked crew without sustaining any injury. About half a gallon of oil was used by the boat during her trip.

The brig P. M. Tenker, Captain Charles Barnard, New York to Cuba, in 1872, encountered a northeast gale when four days out. Several heavy seas came on board, doing great damage. A small bag, with holes punched in the bottom, was filled with oil and hung over the stern. The oil prevented the seas from combing, and the vessel ran for several hours with dry decks.

